# Classification of Parkinson’s disease utilizing multi-edit nearest-neighbor and ensemble learning algorithms with speech samples

**DOI:** 10.1186/s12938-016-0242-6

**Published:** 2016-11-16

**Authors:** He-Hua Zhang, Liuyang Yang, Yuchuan Liu, Pin Wang, Jun Yin, Yongming Li, Mingguo Qiu, Xueru Zhu, Fang Yan

**Affiliations:** 1 Institute of Surgery Research, Daping Hospital, Third Military Medical University, Chongqing, 400042 China; 2 College of Communication Engineering, Chongqing University, Chongqing, 400044 China; 3 Department of Medical Image, College of Biomedical Engineering, Third Military Medical University, Chongqing, 400038 China

**Keywords:** Classification of Parkinson disease, Optimal selection of speech samples, Multi-edit-nearest-neighbor algorithm (MENN), Ensemble learning, Random forest (RF), Decorrelated neural network ensembles (DNNE)

## Abstract

**Background:**

The use of speech based data in the classification of Parkinson disease (PD) has been shown to provide an effect, non-invasive mode of classification in recent years. Thus, there has been an increased interest in speech pattern analysis methods applicable to Parkinsonism for building predictive tele-diagnosis and tele-monitoring models. One of the obstacles in optimizing classifications is to reduce noise within the collected speech samples, thus ensuring better classification accuracy and stability. While the currently used methods are effect, the ability to invoke instance selection has been seldomly examined.

**Methods:**

In this study, a PD classification algorithm was proposed and examined that combines a multi-edit-nearest-neighbor (MENN) algorithm and an ensemble learning algorithm. First, the MENN algorithm is applied for selecting optimal training speech samples iteratively, thereby obtaining samples with high separability. Next, an ensemble learning algorithm, random forest (RF) or decorrelated neural network ensembles (DNNE), is used to generate trained samples from the collected training samples. Lastly, the trained ensemble learning algorithms are applied to the test samples for PD classification. This proposed method was examined using a more recently deposited public datasets and compared against other currently used algorithms for validation.

**Results:**

Experimental results showed that the proposed algorithm obtained the highest degree of improved classification accuracy (29.44%) compared with the other algorithm that was examined. Furthermore, the MENN algorithm alone was found to improve classification accuracy by as much as 45.72%. Moreover, the proposed algorithm was found to exhibit a higher stability, particularly when combining the MENN and RF algorithms.

**Conclusions:**

This study showed that the proposed method could improve PD classification when using speech data and can be applied to future studies seeking to improve PD classification methods.

## Background

Parkinson’s disease (PD) is a neurodegenerative disorder of the central nervous system that is characterized by a partial or full loss in motor reflexes, speech, behavior and other vital functions. It is generally observed in elderly people and causes disorders in patient speech and motor abilities (writing, balance, walk, etc.) [1]. PD is one of the most common neurodegenerative disorders, with an incidence rate of 20/100,000 and affecting approximately 5 million people worldwide, with half of PD cases found in China [2]. Moreover, these statistics are likely to be underestimating the incident rate due to difficulties in diagnosing PD. With populations growing, the number of diagnosed PD patients will continue to grow, thus increasing the damage of this disease in the future [2].

While identifying the causes of PD onset remain elusive, PD often referred to as an idiopathic disorder, genetic and environmental factors have been implicated [1]. Unfortunately, a reliable PD biomarker has yet to be identified, but the symptoms can often reflect PD occurrence and progression, such as tremor, rigidity, loss of muscle control, slowness in movement, poor balance and, especially, voice problems [1–6]. Therefore, diagnosing PD based on symptoms is reasonable and effective [7–14]. Among them, speech has been shown to be a useful signal for discriminating PWP (people with Parkinson’s) from healthy controls, with clinical evidence suggesting that the vast majority of PWP typically exhibit some form of vocal disorder [3, 7, 14–16]. In fact, vocal impairment may be among the earliest prodromal PD symptoms and can be detectable up to five years prior to clinical diagnosis [2]. Thus, utilizing speech data can aid in the development of a noninvasive early PD diagnostic method, with speech alterations including reduced loudness, increased vocal tremor and breathiness (noise), while PD-associated vocal impairment is characterized by dysphonia (inability to produce normal vocal sounds) and dysarthria (difficulty in pronouncing words). Dysphonia can be measured by utilizing acoustic tools that detect voice abnormalities, such as aperiodic vibrations, non-Gaussian randomness, abnormality of vowel “a” phonations, etc. [2], with certain special words and sentences, such as Arabic numerals, special words, etc., used for detection.

While a reliable PD diagnosis is difficult, the effectiveness of speech based diagnostic approaches has inspired researchers to develop decision support tools able to extract dysphonic speech features and design classification algorithms to distinguish PD patients from healthy ones, with speech feature extraction, feature selection/transformation and classifier design [17–24].

When examining feature extraction, the feature types commonly include pitch type, energy type, speed type and content type [1, 2, 17–25]. As for feature transformation, the frequently used algorithms are PCA (principle component analysis) [26, 27, 31, 49]. As for feature selection, the frequently used algorithms are NN (neural network) based [27–30, 32, 49], serial search based [2, 14, 29, 31], random based [32, 33, 48], *p* value based [2, 27–34], relevance based [35, 36] or entropy based [37], discrimination algorithm (DA) based [47]. As for classifier design, the predominantly used classifiers include a support vector machine (SVM) [1, 2, 14, 29, 32, 35, 38–41], KNN [1, 2, 26, 28, 40, 41, 47, 48, 49], random forest (RF) [2, 30, 36], Bayesian network [27, 28, 40, 42, 43, 48], discrimination algorithm (DA) [27, 29, 31, 37], probabilistic neural network (PNN) [27, 43] or decision tree [31, 40, 42, 44–46]. Besides, several ensemble models were involved compared with single classifier [27, 47, 48, 50, 51].

While the methods mentioned above have been effective to some extent, obtaining optimal speech samples is difficult, with low quality samples being prone to misleading the classifiers and negatively impacting accuracy. Based on this potential pitfall, this study examined a speech sample selection algorithm to improve outcomes. First, a multi-edit-nearest-neighbor (MENN) algorithm was utilized to select the optimal speech samples iteratively, thereby enhancing the separability of the training samples. The MENN algorithm effectively optimizes training samples by removing samples that could mislead the classifier [50, 52, 54]. Next, an ensemble learning algorithm, random forest (RF) or decorrelated neural network ensembles (DNNE) was employed for classification on the selected training samples. These two ensembles were chosen for point of comparison, with RF being a classical ensemble learning algorithm with a proven stability [2, 34, 41], while DNNE is a newer ensemble learning algorithm able to effectively maximize differences between sub-learning algorithms [51]. Lastly, a classification was conducted on the test samples based on the trained ensemble learning algorithms.

## Methods

### Data descriptions

The data utilized in this study was obtained from the Parkinson speech dataset with multiple types of sound recordings [1], was deposited by Sakar et al. [1] and is available on the University of California, Irvine (UCI) machine learning dataset repository website. The deposited dataset (Table 1) included two datasets entitled “Training_Data” and “Test_Data”. The “Training_Data” set included 40 subjects, which were 20 PD (6 women, 14 men) and 20 healthy subjects (10 women, 10 men), with 26 speech samples collected per subject. The samples contained a variety of speech segments with varying pronunciations, to include continuous vowel sounds, digital pronunciation, word pronunciation and phrase sentence pronunciation. To set-up a feature vector, linear and nonlinear feature parameters were extracted for each speech sample in 26 dimensions. The “Test_Data” set included 28 subjects (all PD) with six speech samples collected per subject, with half containing pronunciation of the continuous vowel ‘a’, while the other half had pronunciation of the continuous vowel ‘o’. Feature vectors were then constructed by the 26 dimensions.

**Table 1:** Description of speech samples based on the same subject

No of speech samples	Description of speech samples
1st	Sustained vowel of aaa
2nd	Sustained vowel of ooo
3rd	Sustained vowel of uuu
4th–13th	Numbers from 1 to 10
14th–17th	Short sentences
18th–26th	Words

The data collected in the context of this study belongs to 20 PWP (6 female, 14 male) and 20 healthy individuals (10 female, 10 male) who appealed at the Department of Neurology in Cerrahpasa Faculty of Medicine, Istanbul University. Test group consists of patients who are suffering from PD for 0 to 6 years. Individual ages vary between 43 and 77 (mean: 64.86, standard deviation: 8.97) along with 45 and 83 (mean: 62.55, standard deviation: 10.79) for test and control groups, respectively [1].

In addition to the dataset deposited by Sakar et al. [1], another broadly studied dataset also exists that was deposited by Little et al. [2, 14]. The major reasons the Sakar et al. dataset was chosen is as follows: (1) the Little et al. dataset has been more broadly studied, with a classification accuracy over 95% achievable, which is comparable to the close to 100% accuracy achieved herein; (2) the Sakar et al. dataset contains more samples, thus providing more statistically meaningful results; (3) only a few studies have examined the Sakar et al. dataset, thus making findings more insightful; and (4) corresponding classification accuracies for the Sakar et al. dataset are not promising at time. The aim of this study is to show that this type of data collection can lead to high classification accuracy of PD just by altering the classification method. Therefore, in the experimental section, most of the experiments are based on the PD data from Sakar et al. [1]. Since the PD data from Max Little was investigated frequently, the proposed algorithm is verified based on the data too.

### Flowchart of the proposed algorithm (PD_MEdit_EL)

**Fig. 1:**
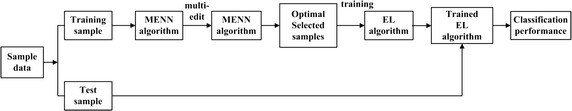
Flowchart of the PD_MEdit_EL algorithm

For simplification, the proposed algorithm was called PD_MEdit_EL and a flowchart can be seen below (Fig. 1). The PD_MEdit_EL algorithm includes two major parts, with one part optimizing speech samples via MENN while the other performs the classification using the ensemble learning algorithm, with either RF or DNNE used. Chronologically, training speech samples are optimized using the MENN algorithm, these training samples are then processed with the ensemble learning (EL) algorithm and a trained learning model is obtained. Finally, the trained learning algorithm is applied to aid in test sample classification.

### Multi-edit-nearest-neighbor algorithm (MENN)

The MENN algorithm serves as a prototype selection algorithm when samples with different classes have overlapping distributions, the overlapping regions are more likely to be misclassified. Presumably, if the overlapping regions are accurately removed, the ‘noise’ or misleading data can be greatly suppressed. Thus, the remaining samples will better reflect the representative samples for different classes, thereby improving the classification accuracy [52].

The two-edit-nearest-neighbor algorithm mainly includes editing and classification. First, samples are divided into training and validation samples, with the validation samples classified and the misclassified samples removed. Generally, if the sample size is large enough, MENN should be applied. The main process of the algorithm is as follows:

*Step 1:* The original samples $X = \left\{ {x_{i} = \left( {x_{i1} ,x_{i2} , \ldots ,x_{id} } \right)|i = 1,{ 2}, \ldots N} \right\}$ were randomly divided into *s* subsets, $X_{ 1} ,X_{ 2} , \ldots ,X_{s} \left( {{\text{s}} > 3} \right)$, each containing $M_{ 1} ,M_{ 2} , \ldots ,M_{s}$ samples. *d* is the dimension of the sample, *N* is the number of samples.

*Step 2:* Editing process. $X_{i + 1}$ is used as the training set and KNN is used to classify the validation samples in set *X*_*i*_, with misclassified samples deleted. These set are repeated for *i* = 1 to *i* = *s* − 1. If *i* = *s*, *X*_1_ is the training set.

*Step 3:* The samples retained after step 2 are merged to form a new sample set (*X*_*New*_).

*Step 4:* Repeat the above steps until all misclassified samples are removed.

**Fig. 2:**
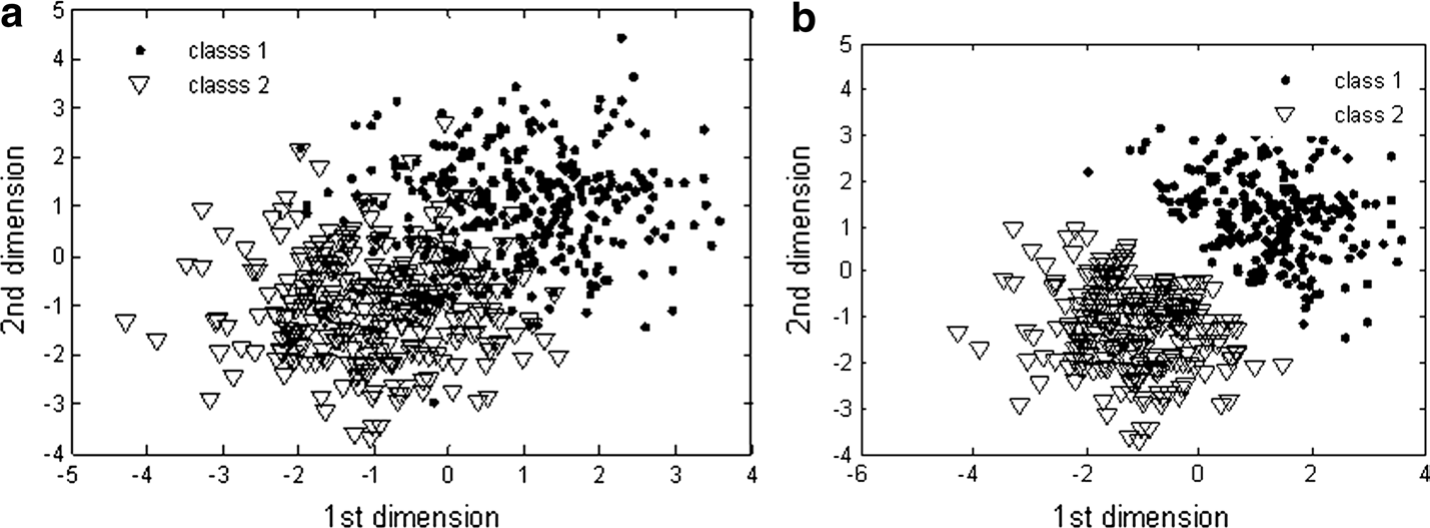
Edited results of training samples: **a** prior to MENN and **b** with MENN.(1st dimension means the 1st feature; 2nd dimension means the 2nd feature of the PD data)

In Fig. 2, the results of the training samples being edited with the MENN algorithm can be seen, with the separability of on the training samples improved when comparing pre-MENN editing (Fig. 2a) and post-MENN editing (Fig. 2b).

Previous studies have shown that the nearest neighbor algorithm can achieve a classification error rate close to the Bayesian error rate P* when a large enough sample size is utilized [53]. For the two-edit-nearest-neighbor algorithm, the progressive conditional false recognition rate is as follows:

1 $$P\,(e/x) = 1 - \frac{{p(\omega_{i} /x)^{2} }}{{\sum\nolimits_{i = 1}^{2} {p(\omega_{i} /x)^{2} } }} .$$

When the MENN algorithm is applied, the progressive conditional false recognition rate is as follows:

2 $$P_{M} (e/x) = 1 - \frac{{\sum\nolimits_{i = 1}^{2} {p(\omega_{i} /x)^{{2^{M + 1} }} } }}{{\sum\nolimits_{i = 1}^{2} {p(\omega_{i} /x)^{{2^{M} }} } }}$$

As seen in the formula, the progressive conditional false recognition rate will be greatly reduced when *M*increases. If $M \to \infty ,{\text{ then lim}}_{M \to + \infty } P_{M} \left( {e/x} \right) = { \hbox{min} }\left[ {p\left( {\omega_{ 1} /x} \right),p\left( {\omega_{ 2} /x} \right)} \right] = P*\left( {e/x} \right)$.

### Random forest

When the RF algorithm is utilized as the classifier, the corresponding training is conducted as follows [54]:

*Step 1:* Bootstrap method is used to re-sample and randomly generate T training sets: $S_{1} ,\;S_{2} , \ldots S_{T}$.

*Step 2:* Corresponding decision trees are generated for each set: $C_{1} ,\;C_{2} , \ldots C_{T}$ and *m*attributes are randomly selected from M attributes, with the determined optimal splitting used to split the node.

*Step 3:* Each tree is allowed to grow integrally, without being pruned.

*Step 4:* Based on each decision tree, the sample in the test set X is classified to generate the corresponding classes: $C_{ 1} \left( X \right),\;C_{ 2} \left( X \right), \ldots C_{T} (X)$.

*Step 5:* The voting method is applied, output categories are determined based on the T decision trees and the category with the maximum votes becomes the assigned category.

### Dnne

The main principle of the DNNE algorithm [51] is described as follows.

*Initialization* Training set*D*_*t*_; base function G (option: sigmoid function); regularizing factor *λ* ∈ [0, 1]; the number of the sub-classifiers is M, dimension of sample is L.

*Procedure* Trained DNNE model.

1: Construct a M single-layer feedback neural network and an ensemble learning algorithm (model).

2: Randomly initialize weights *w*_*j*_ and bias *b*_*j*_.

3: Input training samples *D*_*i*_ and calculate the output *g*_*ij*_(*x*_*n*_) of all the sub-classifiers,

3 $$g_{ij} (x_{n} ) = G_{ij} (w_{j} ,b_{j} ,x_{n} )$$

where the functional connection network (RVFL) for constructing random vector needs training samples *D*_*t*_ with N dimensions, $D_{t} = \left\{ {\left( {x_{1} ,y_{1} } \right),\left( {x_{2} ,y_{2} } \right), \ldots ,\left( {x_{N} ,y_{N} } \right)} \right\}$ and where $\left( {x_{n} ,y_{n} } \right) \in R^{d} \times R$ belongs to pairs of observations. The *G*_*ij*_ () means the *ij* sub-classifier.

4: Calculate constants *C*_*1*_ *, C*_*2*_ as Eq. (4):

4 $$\varphi \left( {i,j,k,l} \right) = \sum\limits_{n = 1}^{N} {g_{ij} \left( {x_{n} } \right)g_{kl} \left( {x_{n} } \right),\quad \varphi \left( {i,j} \right) = \mathop \sum \limits_{n = 1}^{N} g_{ij} \left( {x_{n} } \right)y_{n} }$$

$\varphi \left( {i,j,k,l} \right)$ represents the correlation between the *j*th hidden neuron of *ith* independent RVFL network and the *l*th hidden neuron of *k*th independent RVFL network. $\varphi \left( {i,j} \right)$ signifies the correlation between the *j*th hidden neuron of *i*th based network and object function *ψ*(*x*). Thus, *C*_1_ and *C*_2_ can be represented as Eqs. (5) and (6):

5 $$C_{1} = 1 - 2\lambda \frac{{(M - 1)^{2} }}{{M^{2} }},\quad C_{2} = 2\lambda \frac{M - 1}{{M^{2} }},$$

6 $$\mathop \sum \limits_{k = 1}^{L} C_{1} \varphi \left( {i,j,i,k} \right)\beta_{ik} + \mathop \sum \limits_{l \ne i}^{M} \mathop \sum \limits_{k = 1}^{L} C_{2} \varphi \left( {i,j,l,k} \right)\beta_{ik} = \varphi \left( {i,j} \right).$$

By following the formula above, the error*e*_*i*_, output weight *β*_*ij*_ and *M* × *L* linear equation can be obtained.

So, the linear system can be calculated based on the*β*_*ij*_and the RVFL ensemble model can be obtained. In order to simplify the calculation, the matrix form of the linear system is as follows Eq. (7):

7 $$H_{corr} B_{ens} = T_{h} .$$

*H*_*corr*_ is hidden correlation matrix, *B*_*ens*_ is the global output weights matrix and *T*_*h*_ is the hidden-target matrix. *H*_*corr*_ is defined as follows Eqs. (8) and (9):

8 $$H_{corr} = \left[ \begin{array}{ccccccc} C_{1} \varphi (1,1,1,1) & \cdots & C_{1} \varphi (1,1,1,L) & \cdots & C_{2} \varphi (1,1,M,1) & \cdots & C_{2} \varphi (1,1,M,L) \\ \vdots& & \vdots& & \vdots & & \vdots \\ C_{1} \varphi (1,L,1,1) & \cdots & C_{1} \varphi (1,L,1,L)& \cdots & C_{2} \varphi (1,L,M,1) & \cdots & C_{2} \varphi (1,L,M,L) \\ C_{2} \varphi (2,1,1,1) & \cdots & C_{2} \varphi (2,1,1,L)& \cdots& C_{2} \varphi (2,1,M,1) &\cdots & C_{2} \varphi (2,1,M,L) \\ \vdots & &\vdots & & \vdots & & \vdots \\ C_{2} \varphi (2,L,1,1)& \cdots & C_{2} \varphi (2,L,1,L) & \cdots & C_{2} \varphi (2,1,M,1) & \cdots & C_{2} \varphi (2,L,M,L)\\ \vdots & & \vdots & & \vdots & & \vdots \\ C_{2} \varphi (M,1,1,1) & \cdots &C_{2} \varphi (M,1,1,L) & \cdots & C_{1} \varphi (M,1,M,1) &\cdots & C_{1} \varphi (M,1,M,L) \\ \vdots & & \vdots & & \vdots & & \vdots \\ C_{2} \varphi (M,L,1,1) & \cdots& C_{2} \varphi (M,L,1,L) & \cdots & C_{1} \varphi (M,L,M,1)& \cdots & C_{1} \varphi (M,L,M,L) \end{array} \right]_{(ML \times ML) ,}$$

9 $$H_{corr(p,q)} = \left\{\begin{array}{l} C_{1} \varphi (m,n,k,l)\quad {\text{ if m}} = {\text{k;}} \\ C_{2} \varphi (m,n,k,l)\quad {\text{ otherwise;}} \\ \end{array}\right.$$

where *p*, $q = 1, \ldots ,M \times L;$ $m = \left\lceil {\frac{p}{L}} \right\rceil$, $n = ((p - 1)\bmod \, L) + 1$; $k = \left\lceil {\frac{q}{L}} \right\rceil$; $l = ((q - 1)\bmod \, L) + 1$; and mod means modular operation. *B*_*ens*_ *and T*_*h*_ can be defined as follows Eq. (10):

10 $$B_{ens} = \left[ {\beta_{11} , \ldots ,\beta_{1L} ,\beta_{21} , \ldots ,\beta_{2L} ,\beta_{2L} , \ldots ,\beta_{M1} , \ldots ,\beta_{ML} } \right]_{ML \times 1}^{T} ,$$

$$T_{h} = \left[ {\varphi \left( {1, \, 1} \right), \ldots ,\varphi \left( {1,L} \right),\varphi \left( {2, \, 1} \right), \ldots ,\varphi \left( {2,L} \right), \ldots ,\varphi \left( {M, \, 1} \right), \ldots ,\varphi \left( {M,L} \right)} \right]_{ML \times 1}^{T} .$$

*β*_*ij*_ can be modified using the optimization technique of gradient descent and is formulated as follows Eq. (11):

11 $$\hat{B}_{ens} = H_{corr}^{ - 1} T_{h} .$$

The DNNE algorithm can be programmed and realized. It’s pseudo code is as follows:

## Results

### Experimental condition

One thousand and forty training samples collected from 40 subjects within the “Train_Data” set, with each composed of 26 dimensional feature parameters, were evaluated with leave one out cross validation (LOO CV). If the majority of subject samples were patient, then the subject was deemed a patient; otherwise, the subject was deemed healthy. To reduce the effect of outlier samples from the same subject since not all samples can reflect speech characteristics equally, a cross validation method called leave-one-subject-out (LOSO) was employed. When completing the DNNE algorithm, the linear exhaustive searching algorithm was used to search for maximum parameter values, with the search range of *M* within [2, 15], the base function L of the RVFL network within [5, 50] and the penalty coefficient γ and boosting threshold value ϕ within [0, 1]. Their step sizes were 0.1 and 0.01, respectively. Additionally, 60% of the training samples are frequently used for bagging and boosting the ensemble. When performing RF, the statistically optimal number of decision trees was 500. The SVM was based on libsvm. When performing the MENN algorithm, the setup parameter (*s*) was based on statistical experiments. For MENN, the *s* = 4. For the weights of the classification model, they are set by supervised training.

In this paper, the experimental operating system platform was the Windows, version 7, 32-bit operating system, and the memory size was 4 GB. The data processing was completed in MATLAB, version 2014a. The SVM, RF and relief algorithms are from the toolboxs under MATLAB environment. The DNNE algorithm is from the official website of MATLAB. Original MENN algorithm is from the file exchange related websites (http://www.pudn.com), but it was modified and combined with the DNNE and RF by us. Other parts are designed by us.

### Performance evaluation criteria

To verify the effectiveness of the proposed algorithm, the classification accuracy, sensitivity and specificity were used as an evaluation standard utilizing the terms *TP*: true positive, *TN*: true negative, *FP*: false positive and *FN*: false negative. Specific formulas are as follows:

$$Accuracy = \frac{TP + TN}{TP + FP + TN + FN}$$

Sensitivity, also called the true positive rate, determines the percentage of accurately identified disease subjects by the equation:

$$Sensitivity = \frac{TP}{TP + FN}$$

Specificity, also called the true negative rate, determines the percentage of accurately identified healthy subjects by the equation:

$$Specificity = \frac{TN}{TN + FP}$$

### Classification performance of the PD_MEdit_EL algorithm

In the present study, PD_MEdit_EL algorithm, which utilized the ensemble learning algorithms RF and DNNE, was examined. Until now, only four studies have examined the Sakar et al. [1] dataset, with two only reporting on the classification accuracy of the validation set [42, 49]. Therefore, only two studies [1, 27] remain that are comparable to the PD_MEdit_EL algorithm examined herein. Currently, SVM is widely applied in the classification of the Parkinson’s disease, thus SVMs with linear and RBF kernel functions were compared to the proposed PD_MEdit_EL algorithm (Table 2). These results showed that the PD_MEdit_EL algorithm provided the most accurate classification in terms of classification accuracy (ACC), sensitivity (SEN) and specificity (SPE), regardless of LOO and LOSO. When examining all classification determinants, the RF (with MENN) was the best overall classification algorithm, with the highest mean SPE of LOSO seen with the DNNE (with MENN) algorithm. When comparing the LOO and LOSO methods, the classification performance of LOSO was higher than LOO, possibly due to LOSO effectively reducing outliers within a subject.

**Table 2:** Classification accuracy for the “Training_Data” set

	Leave-one-out method LOO (%)			Leave-one-subject-out method LOSO (%)		
	Accuracy	Sensitivity	Specificity	Accuracy	Sensitivity	Specificity
DNNE (with MENN)						
Mean	67.71	71.71	64	73.37	71	*75*
Std	0.037	0.018	0.033	0.042	0.056	0.032
Best	68.79	72.83	*70*	85	90	80
RF (with MENN)						
Mean	*70.93*	*73.27*	*67.59*	*81.50*	*92.50*	70.50
Std	0.031	0.0052	0.0063	0.0105	0.0225	0.0211
Best	*76.07*	73.65	68.46	*88*	*97*	*88*
SVM (linear)						
Mean	63.65	63.85	63.46	65	65	65
Std	0	0	0	0	0	0
Best	63.65	63.85	63.46	65	65	65
SVM (RBF)						
Mean	63.08	73.08	57.08	67.5	80	55
Std	0	0	0	0	0	0
Best	63.08	73.08	57.08	67.5	80	55
Method in [1]						
Mean	–	–	–	52.06	54.92	49.22
Best	–	–	–	85	80	90
Method in [27]						
Mean	–	–	–	–	–	–
Best	–	–	–	70, KNN (k = 1) 67.5, KNN (k = 3) 72.5, KNN (k = 5) 77.5, KNN (k = 7) 85, SVM (linear) 87.5, SVM (RBF) 80, Naive Bayes 82.5, Discriminant	80, KNN (k = 1) 75, KNN (k = 3) 70, KNN (k = 5) 80, KNN (k = 7) 85, SVM (linear) 90, SVM (RBF) 80, Naive Bayes 80, Discriminant	60, KNN (k = 1) 60, KNN (k = 3) 75, KNN (k = 5) 75, KNN (k = 7) 85, SVM (linear) 85, SVM (RBF) 80, Naive Bayes 85, Discriminant

DNNE (with MENN) and RF (with MENN): reflect the proposed PD_MEdit_EL algorithm; SVM (with linear): SVM with the linear kernel function; SVM (with RBF): SVM with radial basis function kernel; Method in [1]: classification algorithm from the Ref. [1] and Method in [27]: classification algorithm from Ref. [27]

Overall, these findings show that when MENN is combined with either ensemble learning algorithm, a notable improvement is seen over other classification methods. When examining ACC in the RF (with MENN) method, improved classification was seen when compared to the SVM (linear), increased 16.5%, SVM (RBF), increased 14%, method in [1], increased 29.44% and method in [27], increased 0.3%. When examining accuracy in the DNNE (with MENN), classification improved by 8.37% relative to SVM (linear), 5.87% relative to SVM (RBF) and 21.31% relative to the method in [1], but decreased by 2.5% relative to the method in [27]. As for the SEN for the RF (with MENN) method, a classification improvement of 27.5% was seen relative to the SVM (linear), 12.5% relative to the SVM (RBF), 37.58% relative to the method in [1] and 7% relative to the method in [27]. However, when examining the SEN for the DNNE (with MENN) method, a classification improvement was only seen relative to the SVM (linear), increased 6% and the method in [1], increased 16.08%. These results showed that the RF method was more robust than the DNNE algorithm in terms of classification performance.

**Fig. 3:**
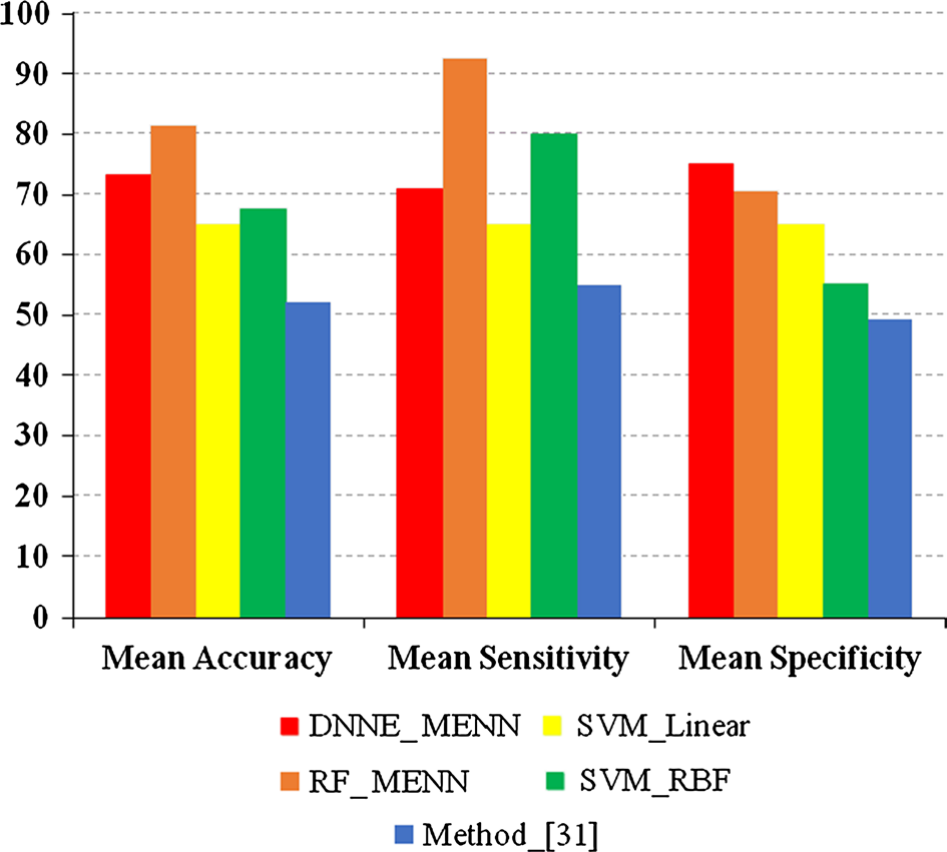
Classification performances between different classification algorithms using the “Training_Data” set. The *y*-axis: classification accuracy; DNNE_MENN and RF_MENN: reflect the proposed PD_MEdit_EL algorithm; SVM_Linear: SVM with the linear kernel function; SVM_RBF: SVM with radial basis function kernel; Method in [27]: classification algorithm from Ref. [27]

**Fig. 4:**
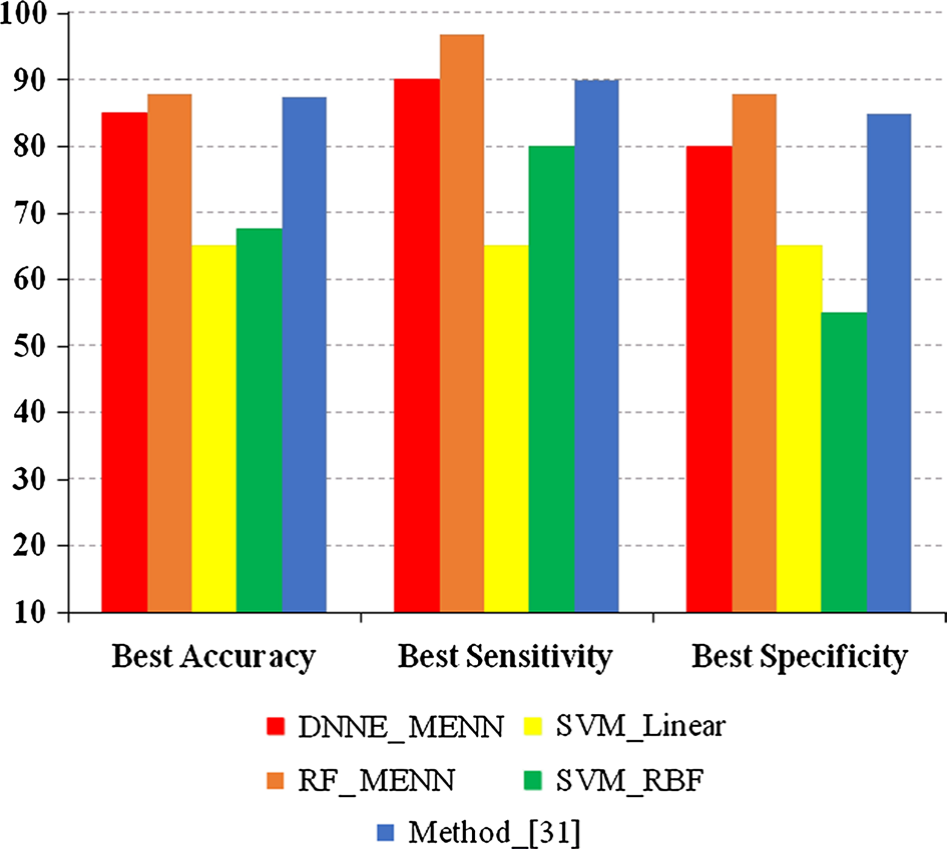
Classification performances between different classification algorithms using the “Test_Data” set. The *y*-axis: classification accuracy; DNNE_MENN and RF_MENN: reflect the proposed PD_MEdit_EL algorithm; SVM_Linear: SVM with the linear kernel function; SVM_RBF: SVM with radial basis function kernel; Method in [27]: classification algorithm from Ref. [27]

Next, the classification accuracy of several classification algorithms was examined for the “Test_Data” set (Table 3). Since this set only contained patient subjects, the sensitivity and specificity could not be calculated. Furthermore, the method in Ref. [27] did not report the classification accuracy for the “Test_Data”, so it was omitted. The results when using this dataset further pointed out the strength of the proposed PD_MEdit_EL algorithm, with significantly higher classification accuracies seen in the RF (with MENN) and DNNE (with MENN). When comparing the DNNE and RF algorithms, no significant difference in terms of classification accuracy was noted. The results obtained in Tables 2 and 3 are also graphically displayed in Figs. 3 and 4.

**Table 3:** Classification accuracy of the “Test_Data” set

	For samples (accuracy) (%)	For subjects (accuracy) (%)
DNNE (with MENN)		
Mean	*95.30*	*98.22*
Std	*0.0101*	*0.0069*
Best	*97.02*	*100*
RF (with MENN)		
Mean	*99.40*	*100*
Std	*0.003*	*0*
Best	*100*	*100*
SVM (linear kernel)		
Mean	68.45	67.86
Std	0	0
Best	68.45	67.86
Method in [1]		
Mean	–	75
Best	–	75

To further examine classification performance, differences between algorithms relative to run number were examined; both the ‘Training_Data’ and ‘Test_Data’ sets were examined 10 times each for each algorithm. These findings were consistent with the data presented in Tables 2 and 3. When examining the “Training_Data” set, both the RF (with MENN) and DNNE (with MENN) showed the highest classification accuracies. However, the RF (with MENN) showed a greater degree of stability compared to the DNNE (with MENN), possibly due to the DNNE being complex. While the DNNE (with MENN) method showed an overall improved classification accuracy, its low stability make it a less ideal candidate. While the method referenced in [1] was not included in this comparison due to the paper [1] did not include the accuracies during number of runs. Since it comprises both SVM_Linear and SVM_RBF components, an indirect comparison can still be drawn to show that the PD_MEdit_EL algorithm provides higher classification accuracy than the method in [1].

**Fig. 5:**
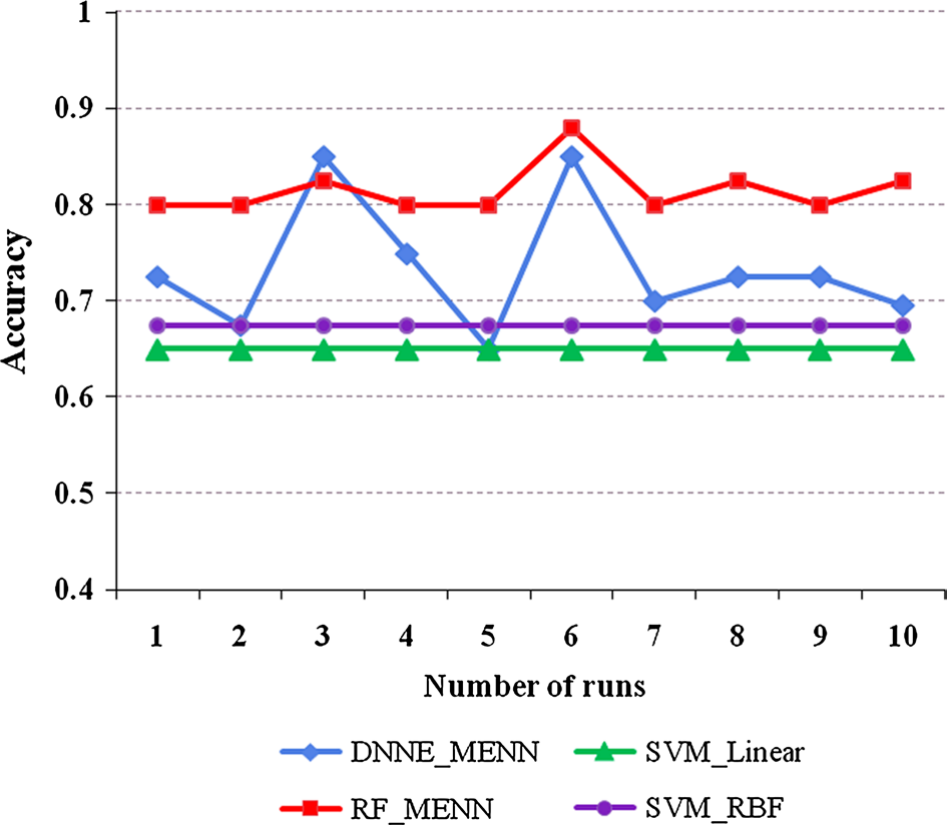
Classification accuracy of different algorithms for the “Training_Data” set. The *y*-axis: classification accuracy; the *x*-axis: the number of runs for each algorithm; DNNE_MENN and RF_MENN: reflect the proposed PD_MEdit_EL algorithm; SVM_Linear: SVM with the linear kernel function; SVM_RBF: SVM with radial basis function kernel

When examining the “Training_Data” set (Fig. 5), the RF (with MENN) again showed the highest degree of accuracy with a high stability, while both the RF (with MENN) and DNNE (with MENN) showed a higher classification accuracy than the SVM, which is currently used in the classification of Parkinson’s disease. When examining this dataset, it is worth noting that the RF becomes more stable, thus suggesting a higher compatibility with this set.

**Fig. 6:**
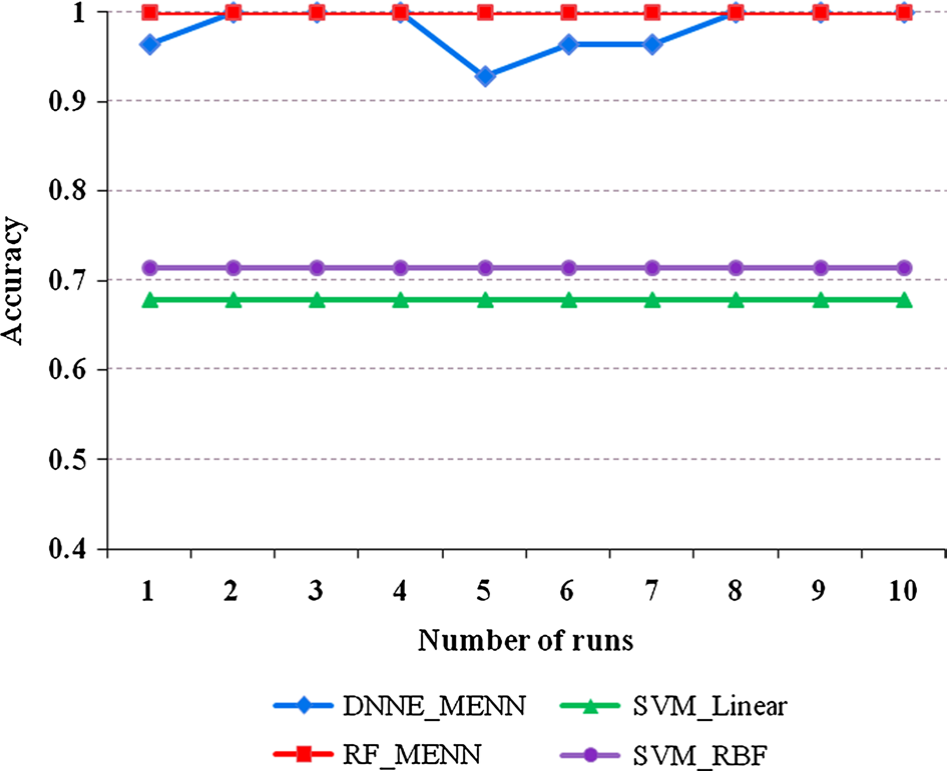
Classification accuracy of different algorithms for the “Test_Data” set. The *y*-axis: classification accuracy; the *x*-axis: the number of runs for each algorithm; DNNE_MENN and RF_MENN: reflect the proposed PD_MEdit_EL algorithm; SVM_Linear: SVM with the linear kernel function; SVM_RBF: SVM with radial basis function kernel

When examining the “Test_Data” set (Fig. 6), the RF (with MENN) again showed the highest degree of accuracy with a high stability, while both the RF (with MENN) and DNNE (with MENN) showed a higher classification accuracy than the SVM, which is currently used in the classification of Parkinson’s disease. When examining this dataset, it is worth noting that the DNNE becomes more stable, thus suggesting a higher compatibility with this set.

The proposed algorithm was verified in the PD data from [2]. The Max Little et al. introduced several machine learning algorithms into their dataset [2]. According to the results, the SVM with RBF kernel and relief algorithm was best. Therefore, it was realized here for comparison with the proposed algorithm. For fair comparison, the proposed algorithm was based on relief algorithm too. The CV method is tenfold CV as same as the paper [2] did.

Seen from the Table 4, the classification accuracy rates are better than those in the Table 2. For the proposed algorithm and LOSO, the classification accuracy is improved from 81.5 to 87.8%; the sensitivity is improved from 92.5 to 95.4% respectively. For the proposed algorithm and LOO, the classification accuracy is improved from 70.93 to 87.8%; the sensitivity is improved from 73.27 to 95.4% respectively. The possible reason is that the number of samples in the dataset is smaller than that in the dataset on the Table 2. Besides, it is worth noting that the sensitivity and the specificity are quite different. The possible reason is that the numbers of the patients and healthy people are different. In other words, the dataset is unbalanced.

**Table 4:** Classification accuracy for the PD data from Max Little et al.

	Tenfold CV (%)		
	Accuracy	Sensitivity	Specificity
RF (with MENN)			
Mean	*87.8*	*95.4*	*65.7*
Std	0.058	0.067	0.175
SVM (RBF)_relief			
Mean	84.5	95.2	52.5
Std	0.061	0.067	0.175

RF (with MENN): reflect the proposed PD_MEdit_EL algorithm; SVM (RBF)_relief: SVM with RBF kernel and relief; SVM (with RBF): SVM with radial basis function kernel; Method in [1]: classification algorithm from the Ref. [1] and Method in [27]: classification algorithm from Ref. [27]

It is worth noting that the 1040 samples are from the 40 subjects, therefore, the samples belonging to same subject are dependent each other to some extent. So, if the samples in training set and test set are independent, the classification accuracy rates possibly become worse. However, the verification section in the relevant papers did not consider this point except the paper the data originates from. In order to further study the problem, an experiment was conducted. In the experiment, when a sample is classified, the other 25 samples belonging to same subject are not used for building the classification model. It can guarantee the samples in training set and test set are independent each other. The Table 5 shows the differences between considering dependency and independency. Seen from the Table 5, the RF_MENN means the algorithm not considering the dependence of the samples; the RF_MENN_inDe means the algorithm considering the dependence of the samples; the SVM_RBF means the SVM with RBF kernel algorithm not considering the dependence of the samples; the RF_MENN_inDe means the SVM with RBF kernel algorithm considering the dependence of the samples.

**Table 5:** Classification result in terms of dependence

	Leave-one-out method LOO (%)			Leave-one-subject-out method LOSO (%)		
	Accuracy	Sensitivity	Specificity	Accuracy	Sensitivity	Specificity
RF_MENN						
Mean	70.93	73.27	67.59	81.50	92.50	70.50
Std	0.031	0.0052	0.0063	0.0105	0.0225	0.0211
Best	76.07	73.65	68.46	88	97	88
RF_MENN_inDe						
Mean	55.1	59.2	51.0	65.0	75.0	55
Std	0.003	0.0063	0.0043	0.0211	0.0242	0.0369
Best	55.4	62.9	53.5	67.5	78.5	59.5
SVM_RBF						
Mean	63.08	73.08	57.08	67.5	80	55
Std	0	0	0	0	0	0
Best	63.08	73.08	57.08	67.5	80	55
SVM_RBF_inDe						
Mean	55.0	62.1	47.8	56.0	71.5	40.5
Std	0	0	0	0	0	0
Best	55.0	62.1	47.8	56.0	71.5	40.5

RF_MENN: reflect the RF + MENN algorithm; RF_MENN_inDe: RF_MENN in the case when a sample is classified and other samples from same subject are not used for building classification model; SVM_RBF: reflect the SVM with RBF kernel; SVM_RBF_inDe: SVM_RBF in the case when a sample is classified and other samples from same subject are not used for building classification model

Seen from the Table 5, when considering the dependence of the samples, the classification accuracy rates become worse regardless of RF and SVM algorithm. The extent of becoming worse is large. However, the proposed algorithm considering dependence of samples is still better than the paper [1]. Compared with the proposed algorithm and the SVM with RBF kernel, the former is still better when considering dependence of samples. It means that the proposed algorithm is valuable.

### Effect of the MENN algorithm

To examine the effects of the MENN algorithm, the “Training_Data” set, which included 20 PD (6 women, 14 men) and 20 healthy (10 women, 10 men) subjects with 26 speech samples per subject, were examined. In total, 1040 speech samples were utilized and examined pre- and post-MENN, with 1039 trained under LOO (Table 6). With the MENN algorithm, the total number of training samples was reduced from 1039 to 731, with the number of healthy subjects reduced from 519 to 364 and the number of patient subjects reduced from 520 to 367. Furthermore, after applying the MENN algorithm, the number of training subjects did not change despite reducing the sample number. Thus, the MENN algorithm can meet the requirement of the subsequent machine learning for number of training samples.

**Fig. 7:**
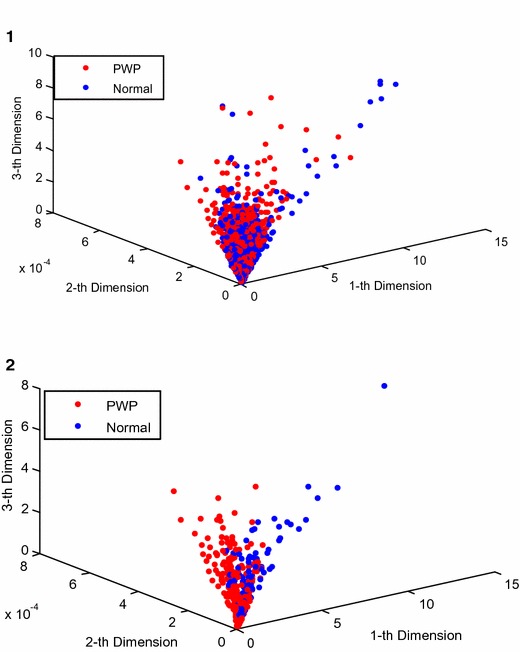
Distribution of samples both (*1*) before and (*2*) after applying the MENN algorithm (1st dimension means the 1st feature of the PD data; 2nd dimension means the 2nd feature of the PD data; 3rd dimension means the 3rd feature of the PD data)

The observed effect of the MENN algorithm was further visualized (Fig. 7), with the three dimensions being the first three features in the datasets and used for coordinate determination. Prior to applying the MENN algorithm, the PWP mean patient samples and normal mean healthy samples were very mixed and difficult to separate (Fig. 7). After apply the MENN algorithm, the sample mixing was greatly improved, thus enabling better subsequent classifications.

Next, an ensemble learning algorithm with and without the MENN algorithm was examined using the “Training_Data” set (Table 7), with the same abbreviations as in Tables 2 and 3 used. The MENN was shown to play an important role in improving classification performance with both DNNE and RF. For DNNE under LOO, the ACC improved 3.62% and the SPE improved 13.33%. For DNNE under LOSO, the ACC improved 4.12%, the SEN improved 2.5% and the SPE improved 5%. For RF under LOO, the ACC improved 3.54%, the SEN improved 1.65% and the SPE improved 5.23%. For DNNE under LOSO, the ACC improved from 3.25% and the SEN improved 7%. The highest improvement was 7% and the highest classification accuracy achieved was 88%.

The ensemble learning algorithms with and without the MENN algorithm were also compared using the “Test_Data” set (Table 8), with the same abbreviations as in Tables 2 and 3 used. These results were in accordance with those presented in Table 5. When examining the ACC, the DNNE for samples improved 14.88%, while the DNNE for subjects improved 10.72%. Also when examining ACC, the RF for samples improved 43.03%, while the RF for subjects improved 45.72%. The highest ACC that was obtained was 100% and the largest increase for the samples was 43.03% and for the subjects was 45.72%. This high degree of improvement may have been due to this dataset only containing patient samples, thus making the classification less difficult.

**Table 6:** Comparison before and after applying the MENN algorithm

Comparison results	Number of training samples (number of subjects)	Number of training samples of healthy subjects (number of subjects)	Number of training samples of patient subjects (number of subjects)
Before applying MENN algorithm	1039 (40)	519 (20)	520 (20)
After applying MENN algorithm	731 (40)	364 (20)	367 (20)

### Level of significance of PD_MEdit_EL algorithm

In an attempt to establish that a significant difference is present between the PD_MEdit_EL algorithm and the other examined algorithms, the p values of the ACCs, SENs and SPEs between the algorithms based on ten times experiments were calculated (Table 9). In terms of LOO and LOSO, the differences between the RF (with MENN) and DNNE (with MENN) when compared to the SVM (linear) or SVM (RBF) showed some highly significant differences. While these results show a significant difference between the PD_MEdit_EL algorithm and those examined, a significant difference was not noted when comparing the RF (with MENN) and DNNE (with MENN), thus suggesting that the two algorithms would perform in a comparable fashion.

**Table 7:** Improvement with the MENN algorithm for the “Training_Data” set

	Leave-one-out LOO (%)			Leave-one-subject-out LOSO (%)		
	Accuracy	Sensitivity	Specificity	Accuracy	Sensitivity	Specificity
DNNE (without MENN)						
Mean	64.09	70	50.67	69.25	68.50	70
Std	0.0178	0.0206	0.0477	0.0274	0.0298	0.0339
Best	66.92	76.71	63.85	75	75	75
DNNE (with MENN)						
Mean	67.71	71.71	64	73.37	71	*75*
Std	0.037	0.018	0.033	0.042	0.056	0.032
Best	68.79	72.83	*70*	85	90	80
RF (without MENN)						
Mean	67.39	71.62	60	78.25	85.50	71.50
Std	0.0141	0.0297	0.0165	0.041	0.035	0.074
Best	68.46	*76.92*	62.36	85	90	85
RF (with MENN)						
Mean	*70.93*	*73.27*	*67.59*	*81.50*	*92.50*	70.50
Std	0.031	0.0052	0.0063	0.0105	0.0225	0.0211
Best	*76.07*	73.65	68.46	*88*	*97*	*88*

Statistically significant differences were also examined using the “Test_Data” set (Table 10). The same trend was seen with this dataset, with some highly significant differences noted between the RF (with MENN) and DNNE (with MENN) when compared to the SVM (linear) or SVM (RBF). However, when comparing the RF (with MENN) and DNNE (with MENN), no significant difference was noted for subjects, but the two were significantly statistically different for samples.

**Table 8:** Improvement with the MENN algorithm for the “Test_Data” set

	For samples (accuracy) (%)	For subjects (accuracy) (%)
DNNE (without MENN)		
Mean	80.42	87.5
Std	0.0327	0.0342
Best	86.9	92.86
DNNE (with MENN)		
Mean	*95.3*	*98.22*
Std	*0.0101*	*0.0069*
Best	*97.02*	*100*
RF (without MENN)		
Mean	56.37	54.28
Std	0.0184	0.0471
Best	59.52	60.71
RF (with MENN)		
Mean	*99.4*	*100*
Std	*0.003*	*0*
Best	*100*	*100*

## Discussion

Herein, a classification algorithm for PD diagnosis, termed PD_MEdit_EL, was generated by combining a multi-edit-nearest neighbor (MENN) algorithm and ensemble learning algorithm. While research pertaining to PD classifications based on speech samples has been performed, few studies have utilized the Sakar et al. dataset [1]. Since this is a larger and more recently deposited dataset, it was chosen to examine the effectiveness of this new classification strategy. While present classification algorithms examine feature extraction, feature selection/transformation and classifier design, they are unable to consider sample optimization via selection. According to the theory of machine learning, the instance selection is very crucial to the quality of machine learning and in accurately obtaining a final classification, with outliers adding ‘noise’ that can mislead the classifier. In the present study, a sample selection algorithm, MENN, is utilized to reduce the impact of these outliers. Subsequent experimentation showed that the proposed algorithm can provide accurate classifications. While the RF method has been utilized in previous studies, a significant improvement was noted when it was combined with the MENN algorithm (Tables 9, 10).

**Table 9:** Establishing method significance using the “Training_Data” set

Significant difference	LOO			LOSO		
	Accuracy	Sensitivity	Specificity	Accuracy	Sensitivity	Specificity
DNNE (with MENN)						
SVM (linear)	<0.0001	<0.0001	0.1888	<0.0001	<0.0001	0.0169
SVM (RBF)	<0.0001	<0.0001	<0.0001	0.0695	<0.0001	0.0207
DNNE (with MENN)						
RF (with MENN)	0.3944	0.9469	0.2596	0.1358	0.1071	0.6319
RF (with MENN)						
SVM (linear)	<0.0001	<0.0001	0.6265	<0.0001	<0.0001	0.0111
SVM (RBF)	0.1454	<0.0001	<0.0001	<0.0001	<0.0001	0.1558

**Table 10:** Establishing method significance using the “Test_Data” set

Significant difference	For samples	For subjects
	Accuracy	Accuracy
DNNE (with MENN)		
SVM (linear)	<0.0001	<0.0001
SVM (RBF)	<0.0001	<0.0001
DNNE (with MENN)		
RF (with MENN)	<0.0001	0.0382
RF (with MENN)		
SVM (linear)	<0.0001	<0.0001
SVM (RBF)	<0.0001	<0.0001

Additionally, two ensemble learning algorithms, RF and DNNE, were utilized to enhance the classification accuracy and stability of classification for PD. While the RF algorithm has been more extensively examined, the DNNE is a new classification algorithm. The parameters for the two algorithms were based on prior knowledge and statistical experiments and the results showed that the two algorithms performed better than the algorithm from Ref. [1], even without the MENN algorithm. When combining the ensemble learning algorithms and the MENN algorithm, the classification performance was further improved in both the “Training_Data” (~30%) and “Test_Data” (~25%) sets. The two Sakar et al. [1] datasets were studied systematically in terms of ACC, SEN and SPE, to include examining samples and subjects. To establish statistical effectiveness of the proposed PD_MEdit_EL algorithm, significance differences between algorithms were examined, with some highly significant differences noted between the PD_MEdit_EL algorithm and the other examined algorithms.

Overall, PD_MEdit_EL algorithm achieved a higher classification performance when compared with examined algorithms currently utilized for classification. This could in part be attributed to the PD_MEdit_EL method processing the data, which the existing examined algorithms do not. Such processing techniques include feature selection, feature nonlinear transformation, multiple-classifier and so on. Thus the combining of these methods makes the PD_MEdit_EL algorithm effective method for classifying PD.

The main contributions and innovations of this paper can be described as follows:

1. Speech samples were optimized utilizing the MENN algorithm, thus improving PD classification accuracy.
2. The ensemble learning algorithms, RF and DNNE, were examined in conjunction with MENN and resulted in further optimized PD classifications.
3. The “Traning_Data” and “Test_Data” sets were studied and verified systematically, while the relevant studies did not involve the “Test_Data” set.
4. The statistical significance of the proposed PD_MEdit_EL algorithm was confirmed when compared to the other examined algorithms.

## Conclusions

While speech based PD classifications have been shown to be effective, the existing methods lack the ability to optimize speech samples, which is crucial for improving PD classification performance. In this study, a new classification algorithm (PD_MEdit_EL) was proposed that combines the MENN algorithm and an ensemble learning algorithm. To evaluate the performance of the proposed algorithm, a public dataset provided by Sakar et al. was utilized, with the proposed algorithm compared to existing methods. Experimental results showed that the PD_MEdit_EL algorithm provides improved classification abilities when compared with the examined algorithms. Furthermore, the noted improvement was apparent irrespective of the LOO or LOSO or of the dataset utilized, with an improvement near 30% seen for the “Training_Data” set and 25% for the “Test_Data”. Overall, this study provides new insight that can be applied to subsequent research pertaining to PD classifications when utilizing speech data. In the near future, we will consider examining compressed speech feature data to further verify and possibly modify the PD_MEdit_EL algorithm.

## Abbreviations

PD: Parkinson disease
MENN: multi-edit-nearest-neighbor
RF: random forest
DNNE: neural network ensembles
PWP: people with Parkinson’s
PCA: principle component analysis
NN: neural network
SVM: support vector machine
PNN: probabilistic neural network
EL: ensemble learning
RVFL: functional connection network LOO
CV: leave one out cross validation
LOSO: leave-one-subject-out
